# Carbon Dioxide Adsorption over Activated Biocarbons Derived from Lemon Peel

**DOI:** 10.3390/molecules29174183

**Published:** 2024-09-04

**Authors:** Karolina Kiełbasa, Joanna Siemak, Joanna Sreńscek-Nazzal, Bestani Benaouda, Banasri Roy, Beata Michalkiewicz

**Affiliations:** 1 Department of Catalytic and Sorbent Materials Engineering, Faculty of Chemical Technology and Engineering, West Pomeranian University of Technology in Szczecin, Pulaskiego 10, 70–322 Szczecin, Poland; karolina.kielbasa@zut.edu.pl (K.K.); joanna.siemak@zut.edu.pl (J.S.); joanna.srenscek@zut.edu.pl (J.S.-N.); 2 Laboratory of Structure, Development, and Application of Molecular Materials (SEA2M), Faculty of Sciences and Technology, Abdelahmid Ibn Badis University of Mostaganem, P.O. Box 227, Mostaganem 27000, Algeria; bestanib@yahoo.fr; 3 Department of Chemical Engineering, Birla Institute of Technology and Science (BITS), Pilani 333031, Rajasthan, India

**Keywords:** CO_2_ adsorption, activated biocarbons, lemon peel

## Abstract

The rising concentration of CO_2_ in the atmosphere is approaching critical levels, posing a significant threat to life on Earth. Porous carbons derived from biobased materials, particularly waste byproducts, offer a viable solution for selective CO_2_ adsorption from large-scale industrial sources, potentially mitigating atmospheric CO_2_ emissions. In this study, we developed highly porous carbons from lemon peel waste through a two-step process, consisting of temperature pretreatment (500 °C) followed by chemical activation by KOH at 850 °C. The largest specific surface area (2821 m^2^/g), total pore volume (1.39 cm^3^/g), and micropore volume (0.70 cm^3^/g) were obtained at the highest KOH-to-carbon ratio of 4. In contrast, the sample activated with a KOH-to-carbon ratio of 2 demonstrated the greatest micropore distribution. This activated biocarbon exhibited superior CO_2_ adsorption capacity, reaching 5.69 mmol/g at 0 °C and 100 kPa. The remarkable adsorption performance can be attributed to the significant volume of micropores with diameters smaller than 0.859 nm. The Radke–Prausnitz equation, traditionally employed to model the adsorption equilibrium of organic compounds from liquid solutions, has been shown to be equally applicable for describing the gas–solid adsorption equilibrium. Furthermore, equations describing the temperature dependence of the Radke–Prausnitz equation’s parameters have been developed.

## 1. Introduction

The augmentation of environmental pollution and the depletion of fossil fuel sources are two very serious threats for the existence of the biosphere as well as human civilization. Environmental pollution is deeply connected to the anthropogenic usage of fossil fuels. According to the *BP Statistical Review of World Energy 2019*, 68th edition data, the global consumption rate of fossil fuels is 13,685 million tons (Mt)/year, with an annual growth rate of 2.8% [1]. In 2020, the global amounts of methane, NO_x_, and carbon dioxide were reported to be 1849.9 ppb, 330.31 ppb, and 410 ppm, respectively [2,3].

According to the *United Nations Environmental Report 2019* [4], the total amount of greenhouse gases in the atmosphere reached 55 gigatons (Gt) and global temperature increased by 3 °C. Hence, the removal of CO_2_ from the environment is crucial. Depending upon the principle of the capture process, there are different types of techniques to separate and capture CO_2_ from the flue gas stream: absorption by means of solvents, membranes, and cryogenics; adsorption by solid materials; capture by microalgae; the calcium looping cycle, etc. Among these, the adsorption of CO_2_, especially at the post-combustion stage, by suitable solid materials with a high surface area such as carbonaceous materials, zeolite, metal–organic frameworks, silica-based materials, etc. could be energetically favorable for industrial applications [5].

Not only as an adsorbent, carbonaceous materials (activated carbon, graphene oxide, CNT, etc.) have attracted great attention due to their wide range of applications such as in energy storage (supercapacitors, batteries, etc.), materials reinforcement, templates for hollow structures, capsules for magnetic nanoparticles, environmental pollution control (both in the gas phase and in solution), drug delivery, etc. [6,7,8,9,10,11].

The improvement in these applications strongly depends on the ability to control the pore size, bulk structure, crystallinity, porosity, and surface properties of the carbon materials. Activated carbons (ACs) are receiving considerable attention for their various impending advantages such as high wear resistance, high mechanical strength, good adsorption performance, high purity, low ash content, high pore volume, and controllable pore size distribution [12,13].

Naturally abundant lignocellulosic biomass wastes (rice hull, corn husk, hazel nutshell, fruit peels, etc.) could be good precursor materials for the synthesis of activated carbon [14,15,16,17,18,19,20,21,22,23,24,25].

These biomass waste resources generate rapidly, are easy to access, and are environmentally benign in nature. Statistically, the European Union produces 7 million tons of orange and other easy-peel citrus fruits [26]. Over the years 2000–2021, the production of lemons and limes increased from 11.38 to 21.55 million tons. However, in 2022, it amounted to 21.53 million tons, which represents a slight decrease compared to the previous year’s production volume [27]. Hence, the production rate of the citrus peel, considered to be roughly 50% of the wet fruit mass, is around 3.5 million tons per year [28].

Several research articles reported the successful production of ACs from lemon peels with high surface areas and porosities.

Divahar et al. [29] developed activated carbon synthesized from lemon peels by the thermal activation method and investigated its lead ion removal efficiency from E-waste bioleachate. The activated carbon had a surface area of 603.7 m^2^/g, a total pore volume of 0.649 cm^3^/g, and an average pore diameter of 0.1 nm.

Weldekidan et al. [30] synthesized highly porous activated carbons from lemon peel waste using a two-step activation process involving hydrothermal carbonization followed by chemical activation with KOH and KOH + aluminum sulfate at 800 °C. The sample activated with KOH + aluminum had the highest BET surface area (2143 m^2^/g) and total pore volume of 1.3 cc/g. While the sample with only KOH activation had a lower surface area (1113 m^2^/g), it had better CO_2_ adsorption capacity, ranging up to 4.5 mmol/g, because of its well-developed pore structures and a high proportion (90%) of micropore distributions.

Yusop et al. [31] synthesized activated carbon from lemon peels by pyrolysis followed by the CO_2_ activation method and Cu salt modification. These materials were used for studying the adsorption of amoxicillin (AMOX) in wastewater. The Cu-modified activated carbon exhibited the maximum BET surface area, pore volume, and pore diameter of 1018 m^2^/g, 0.5377 cm^3^/g, and 2.78 nm, respectively.

De Rose et al. [32] synthesized activated carbon from lemon peels by pyrolysis and the physical activation (in a CO_2_ atmosphere) method, and they studied their hydrogen adsorption characteristics. The maximum surface area and total pore volume were found to be 500 m^2^/g and 0.208 cm^3^/g, respectively.

Li et al. [33] reported on the synthesis of magnetic biochar composite from lemon peel residue by a one-step hydrothermal method, and it was used for the adsorptive capture of methylene blue in an aqueous solution.

Ramutshatsha-Makhwedzha et al. [34] synthesized activated carbon from the 1:1 mixture of waste orange and lemon peel using the phosphoric acid (H_3_PO_4_) activation process and applied it to the removal of Cd^2+^, Pb^2+^, and Fe^2+^ ions from acid mine drainage. The activated carbon had a BET surface area and pore size of 168 m^2^/g and 5.18 nm, respectively.

Ramutshatsha-Makhwedzha et al. [35]) synthesized activated carbon from mixed orange and lemon peels using phosphoric acid and used it for the removal of methyl orange and methylene dyes from wastewater. The BET surface area, pore volume, and pore size of the activated carbon were measured to be 168.29 m^2^/g, 0.269 m^3^/g, and 5.18 nm, respectively.

Praipipat et al. [36] reported on the application of lemon peel beads-doped iron (III) oxide-hydroxide (LBF) and lemon peel beads-doped zinc oxide (LBZ) for the removal of blue 4 dye from water.

Surya and Michael [37] demonstrated the preparation of activated carbon from lemon peels by carbonization at 400 °C followed by chemical activation employing KOH, and the activated carbon was utilized for the fabrication of the electrode of an electrochemical double-layer capacitor.

Naser et al. [38] reported on the synthesis of activated lemon peels using concentrated sulfuric acid and its application for the removal of Pb (II) ions from water.

Su et al. [39] synthesized carbon quantum dots (CQDs) from dried lemon peels using a hydrothermal treatment at 200 °C for a period of 6 h under normal water vapor pressure. The obtained CQDs were utilized for the development of a carmine determination probe sensor.

Sharifzade et al. [40] studied the synthesis of activated carbon from lemon citrus peels using phosphoric acid at 500 °C for the removal of xanthene dyes, erythrosine B (EB), and rhodamine B (RB) from an aqueous binary dye solution.

Mohammadi et al. [41] prepared activated carbon from lemon peels by the chemical activation method using H_3_PO_4_. The BET surface area was revealed to be 1158 m^2^/g.

According to our knowledge, CO_2_ adsorption was investigated over activated carbons derived from lemon peels only once. The lemon peels were converted to activated carbons via hydrothermal carbonization coupled with chemical activation using KOH at 800 °C for CO_2_ adsorption [30]. The KOH–carbon precursor ratio was equal to 2. The specific surface area of 1113 m^2^/g and a CO_2_ adsorption of 4.5 mmol/g at 0 °C and 1 bar were achieved.

Given the very large supply of lemon peel waste worldwide and the limited possibilities for its utilization, we decided to produce activated carbon from this precursor. Moreover, considering the unresolved problem of finding an effective CO_2_ sorbent, we aimed to develop a synthesis of active biocarbon targeted at CO_2_ adsorption. Instead of a hydrothermal pretreatment, we applied a high-temperature pretreatment (500 °C) and demonstrated that our method guaranteed the production of a sorbent with higher CO_2_ adsorption than that obtained by Weldekidan et al. [30], who, like us, also used lemon peels as the carbon precursor and KOH as the activating agent.

The absolute novelty we present here is the application of the Radke–Prausnitz equation to the gas–solid adsorption equilibrium and the demonstration that the parameters of this equation depend on temperature. We also propose forms of these dependencies.

## 2. Results

### 2.1. XRD Results

**Figure 1:**
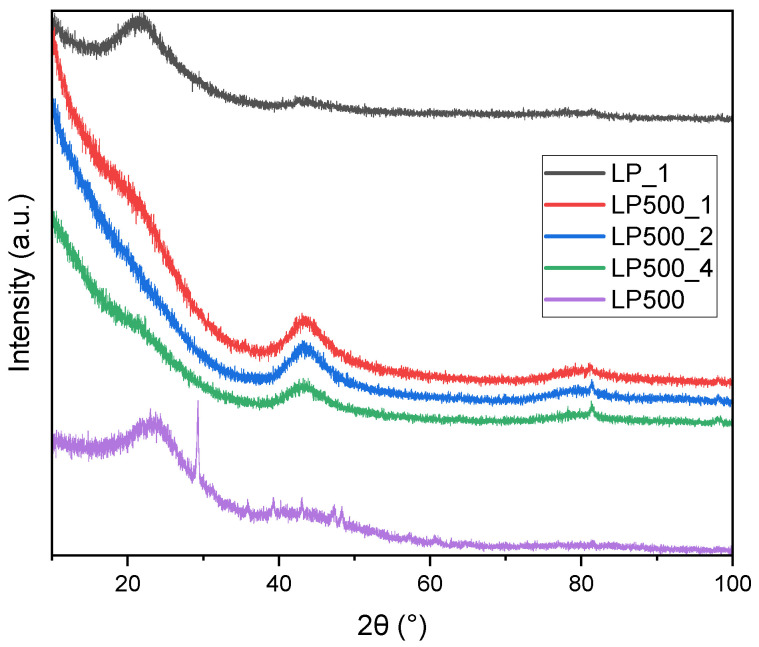
XRD pattern of the activated biocarbons and char derived from lemon peels under different treatment conditions.

The XRD spectra (Figure 1) depict the semicrystalline nature of the activated biocarbons derived from lemon peels under different treatment conditions.

A brief explanation of the activated biocarbons and char derived from the lemon peels under different treatment conditions are presented in Table 1. The detailed explanations are presented in Section 3 of Materials and Methods.

Two prominent peaks at ~23.8° and ~43.6° tally to the (002) and (100) planes (ICSD #76767) of graphitic carbon, respectively. The appearance of these peaks indicates that the activated biocarbons produced from lemon peels were primarily amorphous in nature. After treatment with KOH, the peak at ~23.8° disappeared. This indicates that the individual layers are arranged in a disordered manner relative to each other. However, the signal at ~43.6° became more pronounced. It can be assumed that the average size of the layers increased.

### 2.2. Textural Properties

**Figure 2:**
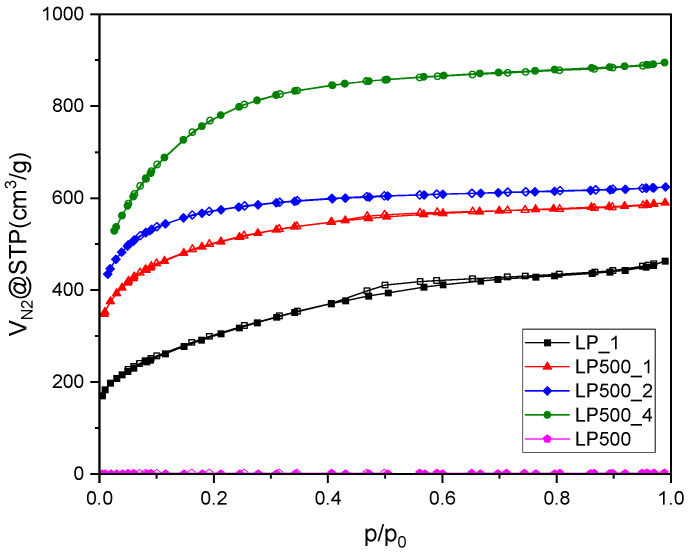
N_2_ adsorption–desorption isotherms acquired at −196 °C for the activated biocarbons and char derived from lemon peels under different treatment conditions.

Figure 2 reveals the N_2_ adsorption–desorption isotherms at −196 °C for the activated biocarbons from lemon peels obtained under different treatment conditions. LP500 showed a very low specific surface area (Table 1) of 0.238 m^2^/g with a total pore volume of 0.003 cm^3^/g. For the pretreated KOH activated biocarbons, after p/p_0_ = 0.3, the adsorption–desorption profiles extended to a flat plateau with the increase in p/p_0_ values generating a classic type I adsorption isotherm, indicating the presence of a very high amount of micropores in the carbon structure. The only KOH-activated sample displayed a slight hysteresis loop between p/p_0_ 0.4 and 0.7, and for the other KOH-treated samples, the adsorption data overlapped with the desorption data.

Other samples showed high N_2_ adsorption, indicating a high BET surface area; however, the biochar pretreatment of the lemon peels significantly enhanced the surface area and pore volume of the activated biocarbon. Lemon peels with only KOH (KOH–lemon peel mass ratio = 1:1) activation (LP_1) had a surface area and pore volume of 1087 m^2^/g and 0.719 cm^3^/g, respectively (Table 2). However, for the sample pretreated at 500 °C followed by KOH activation of same ratio, an almost 80% enhancement of surface area (1835 m^2^/g) and around a 27% enhancement in total pore volume (0.91 cm^3^/g) were observed. In the pretreated samples, these values increased with the KOH–lemon peel mass ratio, and the largest surface area (2821 m^2^/g) and pore volume (1.39 cm^3^/g) were achieved for KOH–lemon peel = 4:1.

When comparing the textural properties of the activated biocarbons from lemon peels activated by KOH produced by us with activated biocarbons obtained from the same carbon source and activated with the same precursor [30], it must be noted that regardless of the amount of KOH used, our materials exhibited higher specific surface area, pore volume, and micropore volume. The key parameter, namely carbon adsorption at 0 °C under 1 bar pressure, was also higher. Most likely, the pretreatment method, specifically the temperature, plays a crucial role.

**Figure 3:**
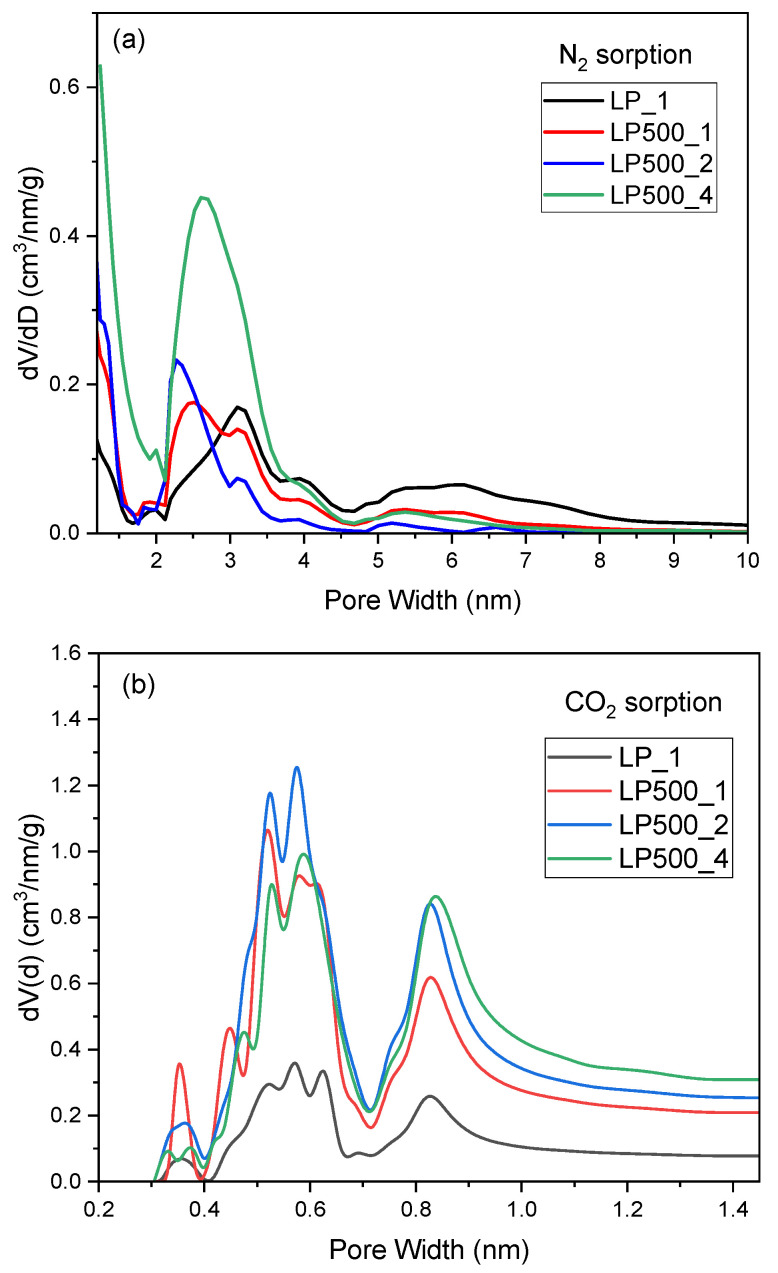
Pore size distribution of the activated biocarbons derived from lemon peels obtained on the basis of N_2_ (**a**) and CO_2_ (**b**) sorption.

Figure 3 shows the pore size distributions of the samples. In the micropore range for materials activated with KOH, the highest pore volume was observed in the ranges of 0.4–0.6 nm and 0.8–0.9 nm. The material LP500_2 had the largest pore volume in the 0.4–0.6 nm range. For the only KOH-activated sample, pores were mostly in the 2–4 and 5–8 nm range, whereas for the pretreated and KOH-activated samples, a large portion of the pores were less than 1 nm and in the 2–4 nm size range.

### 2.3. SEM Results

**Figure 4:**
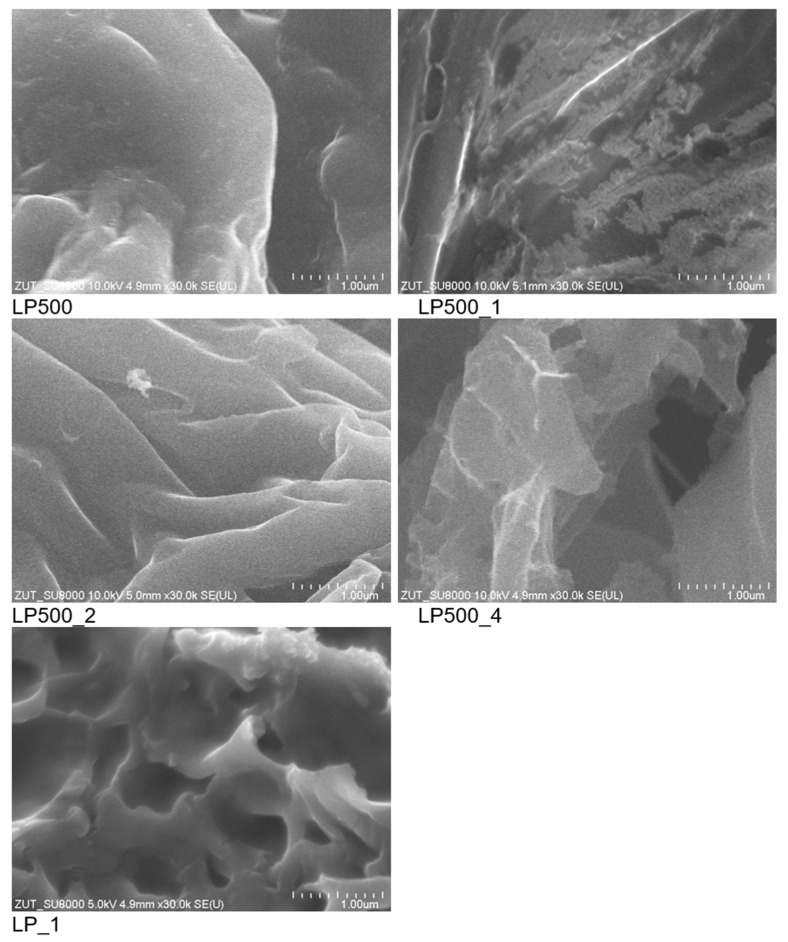
SEM images of activated biocarbons and char derived from lemon peels.

SEM pictures of activated biocarbons and char derived from lemon peels are presented in Figure 4. In the SEM image of the LP500 sample, which was not activated in any way and is simply a carbonized lemon peel, the formation of a non-porous char and a smooth, unwrinkled surface were clearly visible. The SEM images of char that was activated using various amounts of KOH showed increasingly pronounced surface wrinkling as the amount of activator used increased. In the case of using a larger amount of KOH (LP500_4), the surface of the material was composed of thin carbon sheets randomly connected. The material obtained without the preliminary thermal treatment of lemon peels appeared completely different. Treating the carbon precursor with an activating agent and subjecting it to carbonization (LP_1) resulted in a material with a completely different morphology than described above, namely a slightly wrinkled surface characterized by numerous macropores. The SEM images confirm that all chemically activated materials exhibited high porosity, with LP500_4 being the most porous. This is consistent with the N_2_ sorption results presented in Table 1.

### 2.4. CO_2_ Adsorption

**Figure 5:**
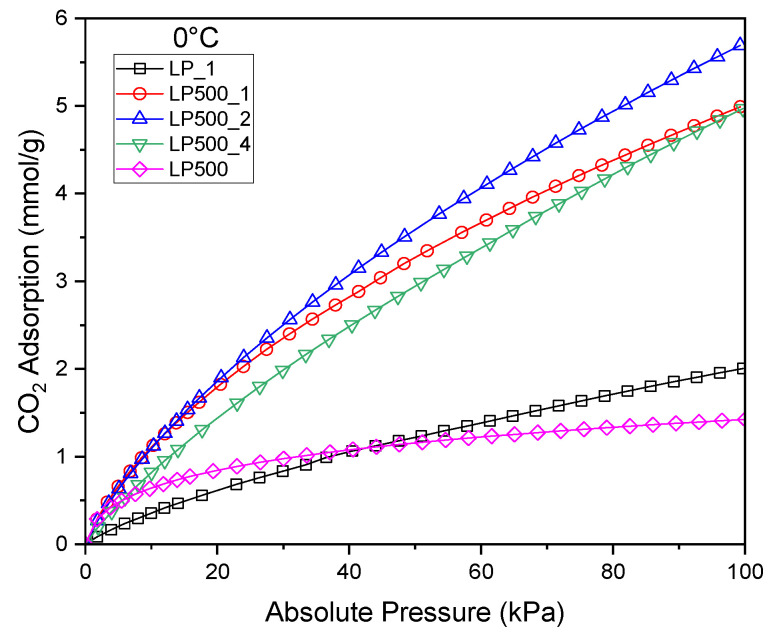
CO_2_ adsorption isotherms up to 1 bar at 0 °C for the activated biocarbons and char derived from lemon peels under different treatment conditions.

Figure 5 shows the CO_2_ adsorption profiles of the activated biocarbons studied at ambient pressure and 0 °C. The maximum CO_2_ uptake (5.69 mmol/g) happened for the pretreated and KOH-activated biocarbon sample with KOH–lemon peel mass ratio = 2:1.

This value is quite high, considering that the synthesis of these activated carbons is relatively simple and inexpensive. If we compare the CO_2_ adsorption values under the same conditions for MOF materials, i.e., 1.67 mmol/g [42] and 3.07 mmol/g [43], whose production is far more complex, it further emphasizes the success of activated biocarbons from lemon peels.

It is important to note here that the maximum surface area and total pore volume were obtained for the pretreated and KOH-activated biocarbon sample with KOH–lemon peel mass ratio = 4:1. However, the high proportion of the micropore distributions (70%) in the pretreated and KOH-activated biocarbon sample with KOH–lemon peel mass ratio = 2:1 (Table 1), along with the narrower pore size distributions (Figure 3), resulted in better CO_2_ adsorption performance of the sample.

**Figure 6:**
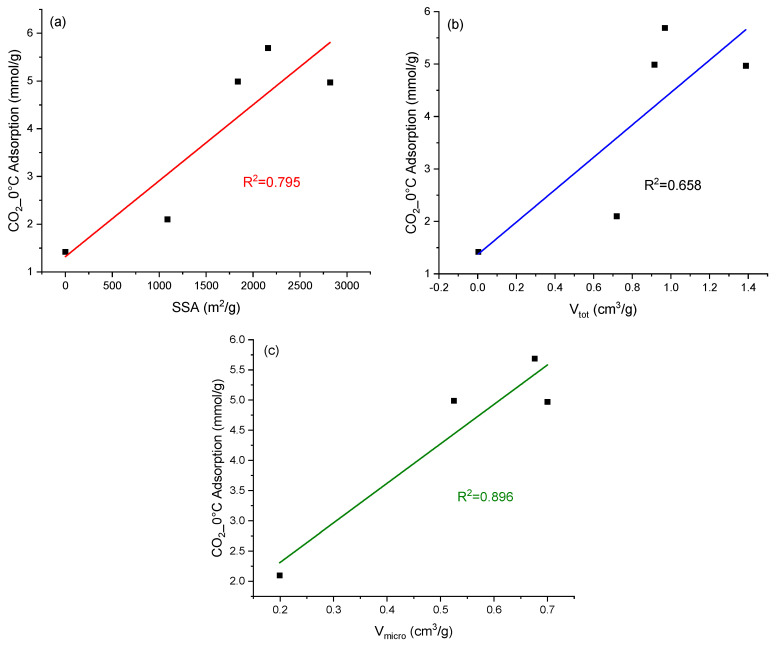
Relationships between CO_2_ adsorption at a temperature of 0 °C and a pressure of 100 kPa, and specific surface area (SSA) (**a**), total pore volume (V_tot_) (**b**), and micropore volume (V_micro_) (**c**).

To better understand the influence of textural parameters on CO_2_ sorption at a temperature of 0 °C and a pressure of 100 kPa, we examined the data presented in Figure 6. Relationships between CO_2_ adsorption at a temperature of 0 °C and a pressure of 100 kPa, and specific surface area (SSA), total pore volume (V_tot_), and micropore volume (V_micro_) show CO_2_ adsorption as a function of specific surface area, total pore volume, and micropore volume.

Casco et al. [44] demonstrated a good correlation between CO_2_ adsorption and surface area, total pore volume, and micropore volume. The R^2^ value was higher than 0.9 for all these textural parameters. Nevertheless, the best correlation (R^2^ = 0.993) was observed for specific surface area. Our studies did not confirm this. The highest, but still unsatisfactory, R^2^ (0.896) value was obtained for micropore volume. The reason may be that the aforementioned authors conducted adsorption studies at a temperature of 25 °C and a pressure of 4.5 MPa. It was shown [45] that, depending on the pressure and temperature conditions, different textural parameters become significant for efficient CO_2_ adsorption.

Considering that in our studies, the R^2^ value suggests that micropores may have some significance for CO_2_ adsorption. We decided to focus on a detailed analysis of the influence of micropore volume with precisely defined diameters.

**Figure 7:**
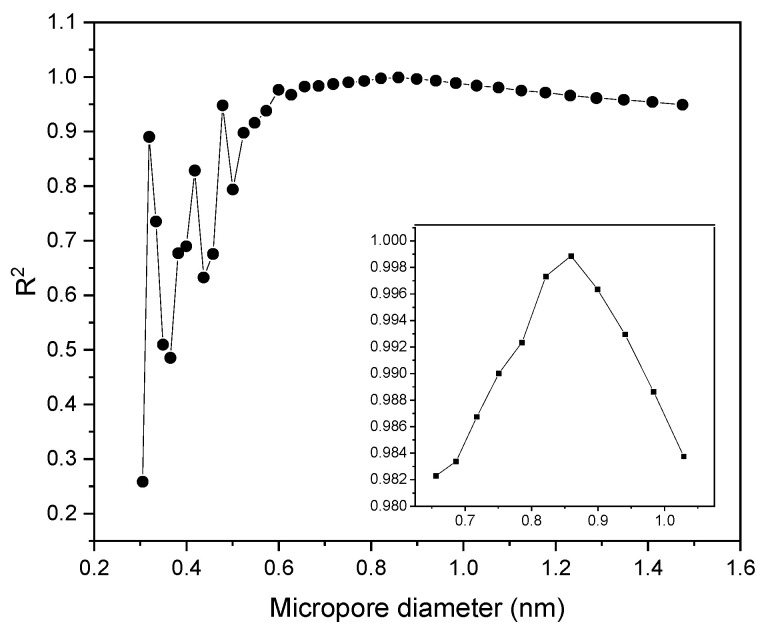
R^2^ values vs. narrow micropore diameter at a temperature of 0 °C and a pressure of 100 kPa.

**Figure 8:**
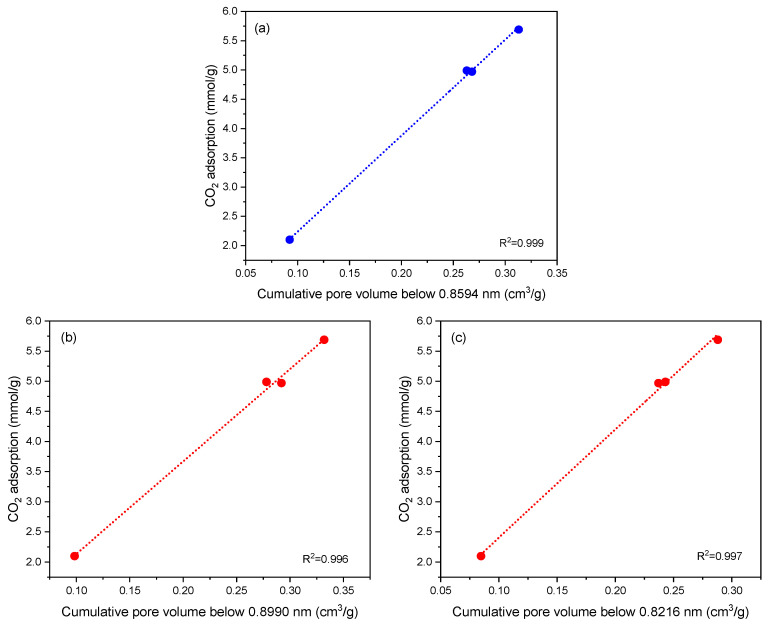
CO_2_ adsorption at a temperature of 0 °C and a pressure of 100 kPa as a function of the volume of pores below 0.8594 (**a**), 0.8990 (**b**) and 0.8216 (**c**) nm.

The relationship between CO_2_ adsorption at 1 bar and 0 °C and the volume of specific pores smaller than a certain diameter was analyzed (Figure 7). The higher coefficient of determination was observed for the volume of pores smaller than 0.859 nm, resulting in an R^2^ of 0.999. The relationships between CO_2_ adsorption and pore diameter for the three highest R^2^ values are presented in Figure 8. The CO_2_ adsorption capacities examined by other authors were primarily attributed to the highly developed microporosity within the range below 0.8 [46,47] and 0.82 [48]. Rouzitalab et al. [45] analyzed over 200 carbon materials and found an apparent discrepancy with the concept that a higher surface area would lead to greater CO_2_ adsorption. In the same comprehensive review, the importance of pore size was highlighted, indicating that the highest CO_2_ adsorption was achieved for materials with a high pore volume in the diameter range of less than 0.8–0.9 nm. Our research, although presented for only four carbon materials, confirms the results reported by other authors.

**Figure 9:**
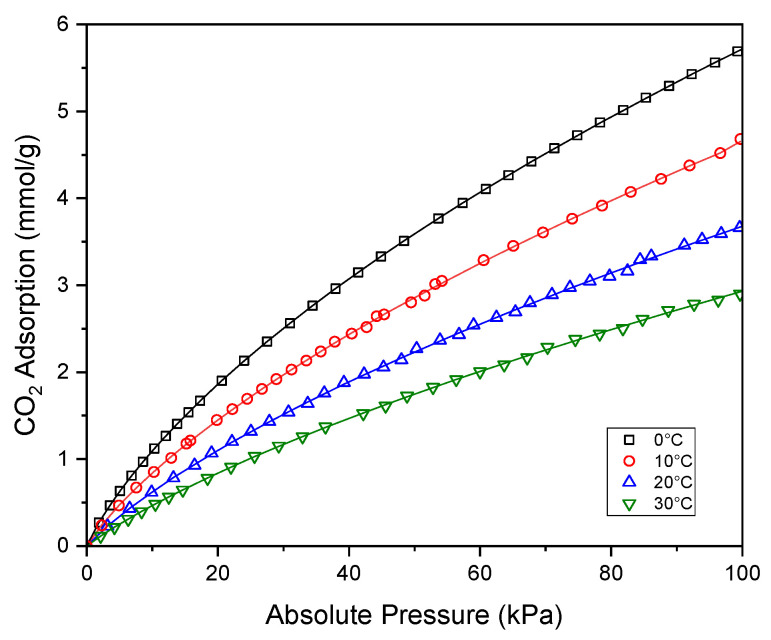
CO_2_ adsorption at temperatures of 0, 10, 20, and 30 °C onto activated biocarbon LP500_2 derived from lemon peels. The points represent experimental data; the lines were drawn using the fitted Radke–Prausnitz model.

The experimental results displayed in Figure 9 were analyzed using the Freundlich, Langmuir, Toth, Sips, UNILAN, and Radke–Prausnitz equations. Five error functions (SSE, HYBRID, ARE, MPSD, and SAE) were applied to find the best-fit model. The Freundlich, Langmuir, Toth, Sips, UNILAN, and error functions were described in detail by Siemak and Michalkiewicz [49]. Since several different equations referred to as Radke–Prausnitz can be found in the literature, it is important to go back to the origins and clarify that the Radke–Prausnitz model was first described by Radke and Prausnitz [50] for the adsorption of organic solutes from a dilute aqueous solution onto activated carbon, and the equation was originally expressed as (1):

(1) $$\frac{1}{{n_{i}}^{c}}=\frac{1}{a{\cdot c}_{i}}+\frac{1}{b\cdot c_{i}^{\beta }}$$

where

*n*_¡_^c^ is the amount of solute adsorbed per unit mass of adsorbent, expressed in millimoles per gram;

*c*_i_ is the solute concentration, expressed in moles per liter;

*ß* is the Radke–Prausnitz parameter restricted to be less than unity.

Authors that later utilized the Radke–Prausnitz equation to other solutions of organic compounds rearranged it to (2) [51,52,53]:

(2) $$\frac{1}{q_{e}}=\frac{1}{a_{RP}{\cdot C}_{e}}+\frac{1}{b_{RP}\cdot C_{e}^{\beta }}$$

By rearranging Equation (2), Equation (3) can be obtained, which represents the nonlinear expression of the Radke–Prausnitz equation [54,55,56].

(3) $$q=\frac{a_{RP}\cdot b_{RP}\cdot C_{e}^{\beta }}{a_{RP}+b_{RP}\cdot C_{e}^{\beta -1}}$$

The common Radke–Prausnitz model used for adsorption from liquid solutions has been adapted by us for gas adsorption. Based on Equation (3), Equation (4) can be derived as

(4) $$q=\frac{a\cdot b\cdot p^{n}}{a+b\cdot p^{n-1}}$$

where:

*q* is CO_2_ equilibrium adsorption at p;

*p* is the equilibrium pressure;

a [mmol/g], b [kPa^−1^], n—Radke-Rrausnitz model constants

Solver, a tool available in Excel, was utilized for finding the parameters and analyzing error functions. The values of the error functions for all the equations listed above are presented in Table 3. The lowest error function values were obtained for the Radke–Prausnitz equation at all temperatures. Among all the models, the Radke–Prausnitz model provided the best fit to the experimental data. Table 4 demonstrate the parameters of the Radke–Prausnitz model and errors of the model.

To effectively describe adsorption equilibrium data across different temperatures, it is essential to have an isotherm equation that accounts for temperature dependence. The temperature dependences of parameters in equations describing adsorption equilibria, such as Freundlich, Langmuir, Sips, Toth, and UNILAN, can be found in the literature [57]. However, such dependencies for the Radke–Prausnitz equation have not yet been described. We have proposed temperature dependencies (Equations (5)–(7)) of the a, b, and n parameters from Equation (4) based on the similarity of the Sips and Toth equations to the Radke–Prausnitz equation:

(5) $$a=a_{0}\cdot exp\left[\chi \left(1-\frac{T}{T_{0}}\right)\right]$$

(6) $$b=b_{0}exp\left[\frac{Q}{RT_{0}}\left(\frac{T_{0}}{T}-1\right)\right]$$

(7) $$n=n_{0}+\alpha \left(1-\frac{T_{0}}{T}\right)$$

**Figure 10:**
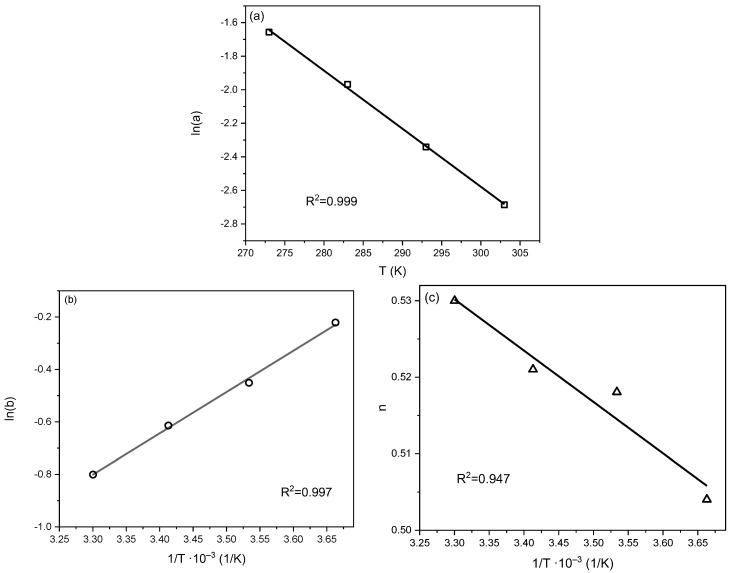
Set of plots of ln(a) vs. T (**a**), b vs. 1/T (**b**), and n vs. 1/T (**c**), plotted for the calculation of a_o_, χ, Q, b_0_, n_0_, and α.

In Equations (5)–(7), a_0_, χ, Q, b_0_, *n*_0_, and α are the constants. R represents the ideal gas constant. T_0_ is the reference temperature. To verify whether the proposed equations can be applied to the Radke–Prausnitz parameters, plots of the natural logarithm of *a* versus temperature, the natural logarithm of *b* versus the inverse of temperature, and *n* versus the inverse of temperature were created and are shown in Figure 10. A temperature of 0 °C was chosen as the reference temperature.

On the basis of the plots presented in Figure 10, the parameters a_0_, α, χ, Q, b_0_, and n_0_ were calculated and presented in Table 5.

The Clausius–Clapeyron equation was employed to calculate the isosteric heat of adsorption:

(8) $$Q_{iso}=-R{\left(\frac{\partial \ln\left(p\right)}{\partial \left(\frac{1}{T}\right)}\right)}_{\theta }$$

where θ is the degree of surface coverage.

After integration of Equation (8), the linear function (9) is obtained:

(9) $${\ln\left(p\right)}_{\theta }=-\frac{Q_{iso}}{R}\frac{1}{T}+C$$

**Figure 11:**
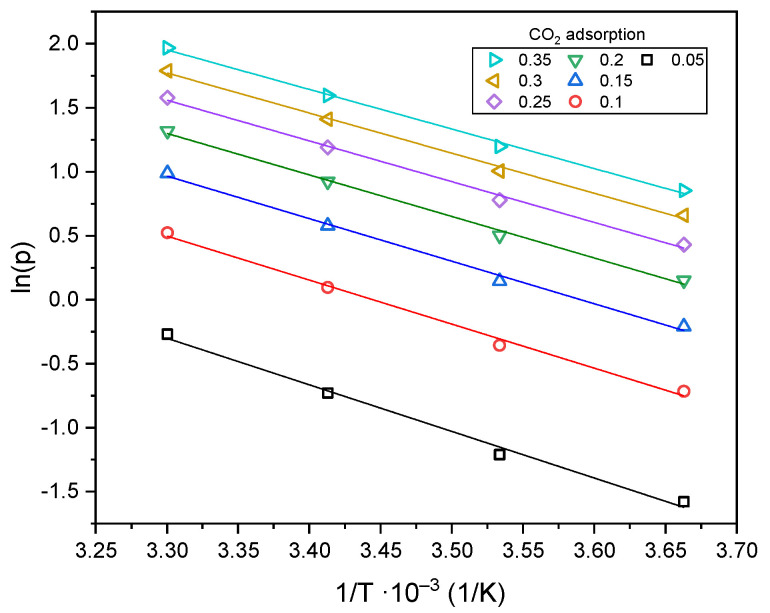
Adsorption isosteres with different surface coverage.

On the basis of the Radke–Prausnitz Equation (4) and the parameters listed in Table 2, the adsorption isosteres (9), i.e., the natural logarithm of pressure values plotted against the inverse of temperature for different fixed surface coverages, were plotted in Figure 11.

**Figure 12:**
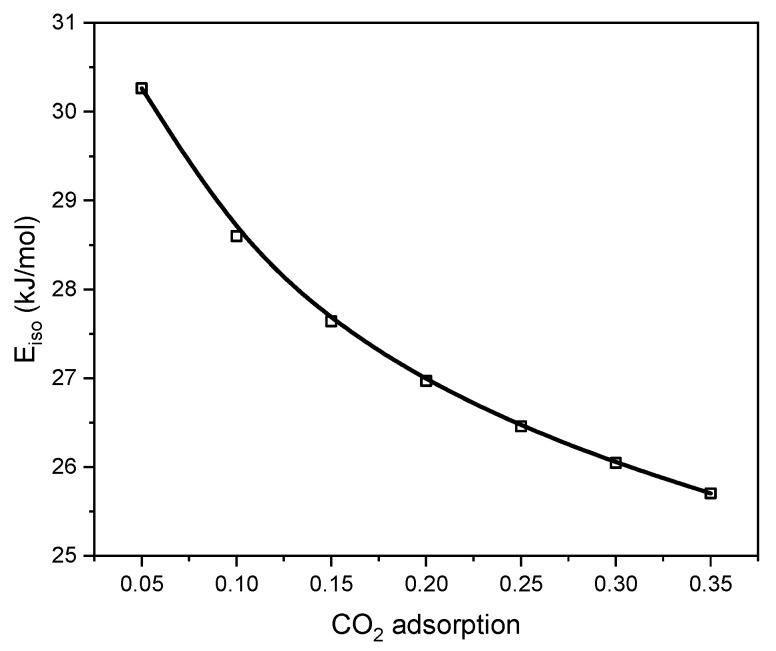
Isosteric heat of CO_2_ adsorption on activated biocarbon LP500_2 derived from lemon peels calculated on the basis of adsorption measurements at temperatures of 0, 10, 20, and 30 °C.

Using the slopes of the resulting straight lines, the isosteric heat of adsorption was determined. The isosteric heat of adsorption at different levels of surface coverage is illustrated in Figure 12.

The isosteric heat of adsorption (Figure 12) was determined through adsorption experiments conducted at the five different temperatures presented in Figure 9. As CO_2_ loading increased, the isosteric heat of adsorption declined. This occurs because the strongest adsorption sites become saturated, leading CO_2_ to be adsorbed onto weaker sites. The calculated values of the isosteric heat of adsorption ranged from 25 to 30 kJ/mol. Such low values indicate that CO_2_ adsorption on activated biocarbon LP500_2 is of a physical nature (physisorption). Suitable adsorbents for CO_2_ capture must, among other things, meet the requirement of gentle regeneration conditions [58]. The ease of regenerating the adsorbent is a crucial factor when selecting materials for CO_2_ separation. A low heat of adsorption ensures that this condition is met. This value of heat adsorption indicates the promise of the activated biocarbon LP500_2 to be used in low-pressure carbon dioxide adsorption applications.

## 3. Materials and Methods

### 3.1. Starting Materials

For the synthesis of activated biocarbon powder from lemon peels, the following chemical reagents were used: potassium hydroxide (Chempur, pure p.a.) and 35–38% hydrochloric acid (Chempur, pure p.a.).

### 3.2. Preparation of Activated Biocarbons from Lemon Peels

Lemons (Citrus limon) were collected from a local market, peels were separated manually, and cut into small pieces of ~0.5 cm × 0.5 cm × 0.5 cm. These were air dried for 48 h and then oven dried at 60 °C for 24 h. The dried peels were crushed in a coffee grinder and strained to collect the powder of ~100–200 µm. The powder was heat treated in two ways. In the first method, it was homogeneously mixed with saturated KOH (KOH–lemon peel mass ratio = 1:1) solution and kept at room temperature for 3 h followed by oven drying at 200 °C for 19 h. The dried mass was carbonized at 850 °C for 1 h under N_2_ flow of 14.4 dm^3^/h. The black mass was collected, transferred to a Buchner funnel, and washed with distilled water until the pH became neutral. The wet black powder was transferred to a 500 cm^3^ beaker containing 100 cm^3^ 1M HCl solution, and the excess solution was evaporated on a hot plate. Again, it was transferred to a Buchner funnel and washed with distilled water until the pH became neutral. Finally, the wet black mass was transferred to a 500 cm^3^ beaker with water, and the excess water was evaporated and dried at 200 °C overnight. The sample was labeled as LP_1. Sample LP500 was prepared similarly but without the addition of KOH.

In the second method, the dried lemon peel powder was first carbonized at 500 °C for 1 h under N_2_ flow of 14.4 cm^3^/h and then impregnated with KOH (KOH–lemon peel mass ratio varied as 1:1, 1:2, and 1:4) and chemically activated in the same way as in the first method. The samples were labeled as LP500_1, LP500_2, and LP500_4. The last symbol refers to the amount of KOH.

### 3.3. Characterization of the Activated Biocarbon

An X-ray diffractometer (X’Pert–PRO, Panalytical, Almelo, The Netherlands, 2012) was used to study the phase of the sample within the range of 2θ = 10–100°. The scanning rate was 0.022349 °/s. The textural properties (specific surface area, pore volume, porosity) of the samples were investigated by a N_2_ adsorption–desorption analyzer (Quadrasorb evo™ Gas Sorption analyzer, Anton Paar, St Albans, UK; previously Quantachrome Instruments, Boynton Beach, FL, USA, 2014). The specific surface area was calculated using the Brunauer–Emmett–Teller (BET) equation from P/P0 = 0.05 to 0.2. The total pore volume was calculated according to the N_2_ adsorbed at P/P0 = 1.

The micropore (1.3–2 nm) volume (V_micro_) was calculated by the DFT method. Total CO_2_ adsorption was determined at 298 K and 1 bar. A field emission scanning electron microscope (SU8020 Ultra-High Resolution Field Emission Scanning Electron Microscope, Hitachi Ltd., Tokyo, Japan, 2012) using secondary and backscattered electron detectors was used to examine the morphology and surface features of the obtained activated biocarbon.

## 4. Conclusions

Lemon peel waste was transformed into highly porous and especially microporous biocarbons through temperature pretreatment (500 °C) followed by chemical activation with KOH at 850 °C, specifically for CO_2_ adsorption. All chemically activated samples had a well-developed micropore structure. The highest specific surface area (2821 m^2^/g), total pore volume (1.39 cm^3^/g), and micropore volume (0.70 cm^3^/g) were achieved for the highest KOH-to-carbon source ratio (4).

The sample activated with a KOH-to-carbon source ratio of 2 exhibited the highest proportion of micropore distributions. This particular activated biocarbon was the best CO_2_ sorbent (5.69 mmol/g at 0 °C and 100 kPa). Such a high adsorption was achieved due to the large volume of micropores smaller than 0.859 nm.

It has been demonstrated that the Radke–Prausnitz equation, previously used to describe the adsorption equilibrium of organic compounds from solutions, can also be applied to describe the gas–solid adsorption equilibrium. Additionally, equations describing the dependence of the Radke–Prausnitz equation parameters on temperature were proposed.

## Figures and Tables

**Table 1:** Brief explanations of the activated biocarbons and char derived from lemon peels.

Sample Name	Thermal Pretreatment	Ratio KOH–Lemon
LP_1	No pretreatment	1
LP500_1	500 °C	1
LP500_2	500 °C	2
LP500_4	500 °C	4
LP500	500 °C	-

**Table 2:** Textural parameters and CO_2_ adsorption at a temperature of 0 °C and a pressure of 100 kPa for activated biocarbons and char derived from lemon peels.

AC	SSA (m^2^/g)	V_tot_ (cm^3^/g)	V_micro_ (cm^3^/g)	V_micro_/V_tot_ (%)	qCO_2__0 °C (mmol/g)
LP_1	1087	0.72	0.20	28	2.01
LP500_1	1836	0.91	0.52	58	4.99
LP500_2	2159	0.97	0.68	70	5.69
LP500_4	2821	1.39	0.70	50	4.97
LP500	0.24	0.003	-	-	1.42

**Table 3:** Error functions values for the Freundlich, Langmuir, Toth, Sips, UNILAN, Radke–Prausnitz equations.

	SSE	HYBRID	ARE	MPSD	SAE
CO_2_ adsorption at 0 °C					
Freundlih	0.066	0.15	2.47	3.66	1.27
Langmuir	0.22	0.41	4.13	5.59	2.43
Sips	0.0023	0.0076	0.56	0.84	0.22
Toth	0.00026	0.00067	0.17	0.24	0.17
UNILAN	0.12	0.24	3.11	4.33	1.81
Radke–Prausnitz	0.00018	0.00013	0.058	0.069	0.061
CO_2_ adsorption at 10 °C					
Freundlih	0.038	0.10	2.18	3.30	0.89
Langmuir	0.11	0.24	3.23	4.71	1.62
Sips	0.009	0.015	0.68	1.00	0.54
Toth	0.0082	0.010	0.51	0.69	0.38
UNILAN	0.068	0.15	2.49	3.73	1.25
Radke–Prausnitz	0.0080	0.0094	0.45	0.65	0.36
CO_2_ adsorption at 20 °C					
Freundlih	0.029	0.077	1.92	2.95	0.82
Langmuir	0.049	0.11	2.34	3.38	1.29
Sips	0.011	0.01	0.68	0.93	0.47
Toth	0.010	0.012	0.57	0.75	0.44
UNILAN	0.033	0.073	1.83	2.70	0.91
Radke–Prausnitz	0.010	0.012	0.55	0.73	0.44
CO_2_ adsorption at 30 °C					
Freundlih	0.019	0.079	2.55	3.52	0.62
Langmuir	0.021	0.073	2.44	3.18	0.69
Sips	0.0057	0.011	0.72	0.87	0.35
Toth	0.0053	0.0082	0.50	0.62	0.30
UNILAN	0.015	0.049	1.94	2.56	0.58
Radke–Prausnitz	0.0052	0.0080	0.48	0.61	0.29

**Table 4:** The parameters of the Radke–Prausnitz model and errors of the model for activated biocarbon LP500_2 derived from lemon peels at different adsorption temperatures.

Temp	a	b	n	SSE	HYBRID	ARE	MPSD	SAE
0 °C	0.191	0.802	0.504	0.00018	0.00013	0.058	0.069	0.060
10 °C	0.140	0.637	0.518	0.0080	0.0094	0.46	0.65	0.36
20 °C	0.096	0.541	0.521	0.010	0.012	0.55	0.73	0.44
30 °C	0.068	0.449	0.530	0.0052	0.0080	0.47	0.61	0.29

**Table 5:** The parameters of temperature-dependent Radke–Prausnitz model equations.

Parameter	Value	Unit
Q	13 090	J/mol
b_0_	0.490	kPa^−1^
n_0_	0.505	
α	0.24	
a_0_	0.193	mmol/g
χ	10.49	

## Data Availability

The original data presented in this study are openly available in Mendeley Data at https://data.mendeley.com/datasets/94jg7jkt6n/1 (accessed on 8 August 2024).
